# The subgenual organ complex in the cave cricket *Troglophilus neglectus* (Orthoptera: Rhaphidophoridae): comparative innervation and sensory evolution

**DOI:** 10.1098/rsos.140240

**Published:** 2014-10-01

**Authors:** Johannes Strauß, Nataša Stritih, Reinhard Lakes-Harlan

**Affiliations:** 1 AG Integrative Sensory Physiology, Institute for Animal Physiology, Justus-Liebig- Universität Gießen, Gießen, Germany; 2 Department of Entomology, National Institute of Biology, Ljubljana, Slovenia

**Keywords:** neuroanatomy, chordotonal organ, mechanoreceptor, sensory innervation, neural phylogeny

## Abstract

Comparative studies of the organization of nervous systems and sensory organs can reveal their evolution and specific adaptations. In the forelegs of some Ensifera (including crickets and tettigoniids), tympanal hearing organs are located in close proximity to the mechanosensitive subgenual organ (SGO). In the present study, the SGO complex in the non-hearing cave cricket *Troglophilus neglectus* (Rhaphidophoridae) is investigated for the neuronal innervation pattern and for organs homologous to the hearing organs in related taxa. We analyse the innervation pattern of the sensory organs (SGO and intermediate organ (IO)) and its variability between individuals. In *T. neglectus*, the IO consists of two major groups of closely associated sensilla with different positions. While the distal-most sensilla superficially resemble tettigoniid auditory sensilla in location and orientation, the sensory innervation does not show these two groups to be distinct organs. Though variability in the number of sensory nerve branches occurs, usually either organ is supplied by a single nerve branch. Hence, no sensory elements clearly homologous to the auditory organ are evident. In contrast to other non-hearing Ensifera, the cave cricket sensory structures are relatively simple, consistent with a plesiomorphic organization resembling sensory innervation in grasshoppers and stick insects.

## 2. Introduction

For several species of insects, the neuronal innervation pattern of legs has been documented with particular emphasis on the complex sensory organs containing numerous sensory neurons [1–4]. Scolopidial sensilla form internal sensory organs distributed over all body segments. They consist of one or more sensory neurons and additional cell types [5–8]. The sensory neurons of scolopidia are activated by stretching of the dendrite and code for a variety of mechanical forces, caused by body tension as well as external stimuli like substrate vibrations or sound [8–12].

The subgenual organ (SGO) is an important scolopidial organ present in the tibia of most insects, which is most sensitive to substrate vibrations [13] but may also respond to airborne sound [14–18]. Among several species of Blattodea and Orthoptera, the SGO is commonly found next to other, closely associated sensory organs also containing scolopidial sensilla [8,19–21]. These additional organs are, for example, the distal organ (DO) [19,22] or the intermediate organ (IO) [23]. Because in most orthopteroid insects more than one sensory organ occurs in the proximal tibia, these are together called the SGO complex [21]. These organs are usually supplied by distinct nerve branches.

Ensifera (the ‘long-horned grasshoppers’) are well studied for their auditory organs (tympanal organs characterized by thin tympanal membranes), located in the foreleg tibia of some taxa [24–26]. In these tympanal organs, specific sets of auditory sensilla occur close to the SGO, which respond to airborne sound with high sensitivity, e.g. in crickets [27–30] and tettigoniids [23,31–33]. In tettigoniids and some related hearing Ensifera, the so-called complex tibial organ consists of the SGO, the IO and the *crista acustica* (table 1). The tettigoniid *crista acustica* contains the auditory sensilla [31,32,43], which respond to airborne sound with a specific frequency tuning of individual sensilla [17,44,45]. The auditory sensilla are usually arranged along the proximo-distal leg axis in a characteristical linear array [23,31,43,46,47]. A morphologically similar hearing organ (the tympanal organ) is present in crickets [28,30,48]. However, the homology of tympanal organs in crickets and tettigoniids is not adequately resolved [23,26,49,50].

**Table 1.** Taxonomic groups of Ensifera with sensory organs in the SGO complex. It is indicated if the taxa are atympanate (AT) or generally tympanate (T), though cases of secondary tympana reduction occur repeatedly among crickets and tettigoniids. The sensory organs are innervated by the main sensory nerve in the leg. Data are from Blattidae: *Periplaneta americana* [22]; Blattidae: *Blaberus discoidales*, *Blattera germanica* [34]; Mantophasmatodea [35]; Phasmatodea: *Carausius morosus*, *Sipyloidea sipylus* [21]; Caelifera: *Schistocerca gregaria* [3]; Caelifera: *Locusta migratoria*, *Schistocerca gregaria* [36]; Tettigoniidae: *Decticus verrucivorus* [23]; Anostostomatidae: *Hemideina femorata* [4]; Anostostomatidae: *Hemideina crassidens* [37]; Haglidae: *Cyphoderris monstrosa* [38]; Stenopelmatidae: *Stenopelmatus spec*. [39]; Gryllacrididae: *Ametrus tibialis* [40]; Rhaphidophoridae: *Troglophilus neglectus* [41]; Gryllidae: *Achaeta domestica* [23]; Gryllidae: *Gryllus bimaculatus* [28]; Gryllidae: *Gryllus bimacultus*, *G. campestris* [30]; Gryllotalpidae: *Gryllotalpa major* [42], and own unpublished observations (2012–2013). AT, atympanate taxon; CA, *crista acustica*; CAH, *crista acustica* homologue; DO, distal organ; IO, intermediate organ; NO, Nebenorgan; SGO, subgenual organ; T, tympanate taxon.

		presence of tibial tympana (T)/	
taxon	common names	absence of tibial tympana (AT)	sensory organs
Blattidae	cockroaches	AT	SGO, DO, NO
Mantophasmatodea	heelwalkers	AT	SGO, DO, NO
Phasmatodea	stick insects	AT	SGO, DO
Caelifera	grasshoppers	AT	SGO, DO
Ensifera: Tettigonioidea			
Rhaphidophoridae	camel, cave and sand-treader crickets	AT	SGO, IO
Gryllacrididae	raspy crickets	AT	SGO, IO, CAH
Cooloolidae	cooloola monster	AT	not investigated
Stenopelmatidae	Jerusalem crickets	AT	SGO, IO, CAH
Schizodactylidae	splay-footed crickets	AT	SGO, IO, CAH
Haglidae	hump-winged grigs or crickets	T	SGO, IO, CA
Tettigoniidae	katydids/bushcrickets	T	SGO, IO, CA
Anostostomatidae	weta	T/AT	SGO, IO, CA
Ensifera: Grylloidea			
Gryllidae	crickets	T	SGO, TO
Gryllotalpidae	mole crickets	T	SGO, TO

Besides these tympanate groups, Ensifera also include atympanate groups that completely lack a tegminal (wing) stridulation apparatus for sound production and tympanal ears in the foreleg (table 1) [25,26,51].

The Rhaphidophoridae or ‘cave crickets’ generally lack auditory tympana or vestiges thereof [24,52] and may be a basal group in the ensiferan lineage [25]. These species occupy a great diversity of niches, most including different degrees of specialization to cave life [53]. *Troglophilus neglectus* uses caves only as a refuge during day time and the winter season, while it forages and reproduces in forests during night time [54,55]. Its mating behaviour includes male-produced vibratory signals and seems to reflect a primitive signalling pattern among Ensifera [56,57]. In accordance with this, the SGO complex of the species consists of the SGO and the IO only [41], and both are sensitive to vibrations transferred to the sensory organs through the leg [58]. The sensory organs respond to airborne sound only of low frequencies at high amplitudes [41]. The IO is anatomically differentiated into a proximal intermediate organ (pIO) and a distal intermediate organ (dIO) with specific arrangements of sensory cells [41]. By contrast, all other atympanate taxa in Ensifera investigated so far include a sensory organ clearly homologous to the *crista acustica* of tettigoniids [51].

The evolution of this structural diversity in the SGO complex and the sensory functions of the different organs across Ensifera are not resolved. In addition to the SGO, another organ is commonly located distally of the SGO. This is termed the DO (Blattidae, Mantophasmatodea, Phasmatodea, Caelifera) or IO (Ensifera), and these organs are not well characterized in terms of their physiology. Since these organs are common among Orthopteroidea and share the position in the anterior tibia, they may actually be homologous [51]. The physiological function of the IO depends on the presence of a tympanal organ. In tettigoniid species with tympanal hearing organs, the IO may respond to airborne sound (up to 8 kHz) [14,17,59]. In atympanate legs of tettigoniids, the IO is insensitive to sound but sensitive to vibrations with a maximum sensitivity between 600 and 1000 Hz [60]. The DO in cockroaches has been suggested to measure changes in the hemolymphe pressure [22] but may contain vibration-sensitive sensilla [13]. Both DO and IO may differentiate into anatomically distinct, proximal and distal sets of sensilla [22,61–64]. The *crista acustica* is the main auditory organ in Tettigoniidae [31,44], Haglidae [38] and Anostostomatidae (wetas) [65]. The physiology of the *crista acustica* homologue in atympanate Ensifera has not been studied in detail but was suggested to be vibration-sensitive, possibly with a different tuning than sensilla in the SGO [40]. A small scolopidial organ, the Nebenorgan [22,35] or accessory organ [37,64], may occur on the posterior side in the leg. In cockroaches, it has been suggested to perceive low-frequency vibrations [22].

With respect to the evolution of sensory organs, it is plausible that the SGO complex in orthopteroid insects ancestrally contained two major organs innervated by the main sensory nerve, the SGO and DO/IO, as these organs are commonly present [51]. In Tettigonioidea, the *crista acustica* homologue or *crista acustica* was added. The highly conserved distribution of the *crista acustica* homologue in atympanate taxa raises the question if the neuroanatomy of the SGO complex in cave crickets is indeed ‘primitive’ compared to the hearing Ensifera [41] and resembles a plesiomorphic organization prior to the evolution of the *crista acustica* homologue, or if it may in fact share this sensory homologue to auditory sensilla with other atympanate Ensifera. Remarkably, the five to six neurons in the dIO of *T. neglectus* occur in a line [41], not unlike the *crista acustica*.

In tettigonioids, each of the scolopidial organs in the SGO complex is usually innervated by a distinct nerve branch [1,4,31,39,64]. In *T. neglectus*, the sensory innervation of the SGO complex has not been documented [41]. Here, we analyse innervation patterns of the sensory organs in *T. neglectus* for their similarity to other tettigonioids. Axonal pathways or innervation patterns of sensory organs are helpful for identification and comparison of neural elements across taxa, as they appear to be rather conserved in evolution [66–70]. Some variability in the innervation pattern can nevertheless be expected between individuals even within one species, as was described from ensiferan SGOs in both tympanate and atympanate species [1,39]. This variability is most probably due to stochastic alterations in axonal pathfinding of sensory neurons during embryogenesis.

In this study, we describe the innervation pattern of the SGO and IO and compare its variability between individuals. The aim is to document whether the pIO and dIO in *T. neglectus* have a joint or separate innervation, supporting them as a single organ or rather two distinct organs. These data are compared to those from other Ensifera. If separate innervations for the pIO and the dIO are confirmed, this might indicate that the dIO sensilla correspond to the *crista acustica* homologue of atympanate Ensifera, thus highlighting the presence of shared sensory structures across the atympanate groups of Ensifera.

## 3. Material and methods

### 3.1 Animals

Animals were caught in northwestern Slovenia, in a cave in the vicinity of Most na Soči. They were maintained in the laboratory in terraria filled with moss, at room temperature and in constant darkness and fed with dried fish food ad libitum. High humidity in the terraria was maintained by keeping the moss moist.

### 3.2 Axonal tracing experiments

Retrograde tracing of both nerves 5B1 and 5B2 was carried out to document the neuroanatomical organization of the tibial organ in *T. neglectus*. The legs were cut off at the proximal femur and mounted in Sylgaard-covered glass dishes (Sylgaard 184, Suter Kunstoffe AG, Fraubrunnen, Switzerland) with insect pins under locust saline [71] (pH=7.2). They were opened ventrally with a piece of a razor blade. Nerves were cut with iridectomy scissors proximal of the femur–tibia joint. In some experiments, only one of the leg nerves 5B1 or 5B2 was filled to show the innervated structures, while in other experiments both were filled to show the whole sensory complex. The cut nerve ends were transferred into a glass capillary filled with 5% CoCl_2_ solution (cobalt chloride from Merck, Darmstadt, Germany) dissolved in distilled water [72,73]. The preparations were then incubated for 48 h at 4°C. For visualization of the fills, the cobalt was precipitated by incubating the legs in a solution of 1% ammonium sulfide (Fluka, Buchs, Switzerland) in locust ringer for 10–15 min at room temperature. The legs were rinsed in locust saline and immediately fixed for 60 min in chilled 4% paraformaldehyde (Sigma Chemicals, St Louis, MO, USA) dissolved in phosphate buffer (0.04 mol l^−1^ Na_2_HPO_4_, 0.00574 mol l^−1^ NaH_2_PO_4_×2H_2_O; pH=7.4). The preparations were dehydrated in a graded ethanol series (Carl Roth, Karlsruhe, Germany) for 60 min at each step, and finally cleared in methyl salicylate (Fluka). Overall, 48 leg preparations were of adequate quality to be analysed for this study. All thoracic leg pairs were included. As previously found [41], there were no differences in the sensory neuroanatomy between organs in different leg pairs.

Anterograde tracing of the nerve 5B1 for sensory projections into the central nervous system was carried out. After the animals were anaesthetized with CO_2_, they were mounted in a Petri dish ventral side up, using insect pins and a beeswax–colophony mixture. The cuticle was removed anteriorly and proximally in the femur. The nerve was cut and placed into a glass capillary filled with NiCl_2_, or Lucifer Yellow (each 5%; Sigma) dissolved in distilled water. After incubation at 4°C for 48 h, the thoracic ganglia were excised and NiCl_2_ was precipitated by the saturated solution of rubeanic acid (Merck) in 100% ethanol, by adding three to five drops to 1 ml of saline for 10 min, fixed in Carnoy's solution (1:4 mixture of glacial acetic acid (Sigma) and 100% ethanol) and silver intensified [74]. All preparations were dehydrated in ethanol series (for 10 min in each step) and cleared in methyl salicylate (Sigma). After the morphological analysis of wholemount preparations (see below), preparations of T1 were embedded in epoxy resin (Agar 100 resin kit; Agar scientific, Stansted, UK), serially sectioned in the transverse plane (18 μm) and embedded in Floromount (Sigma).

### 3.3 Analysis and documentation

The preparations were viewed with an Olympus BH-2 microscope (peripheral innervation) and with a Leica DMRB/E microscope (central projections). Photos of the respective preparations were taken with a Leica DCF-320 camera (2088×1055 pixel) and Zeiss AxioCam MRc camera (1300×1030 pixel), respectively, that was attached to the microscope. Most preparations included here were photographed in series, and stacked pictures were obtained with the freeware program CombineZP (http://www.hadleyweb.pwp.blueyonder.co.uk/CZP/Installation.htm). Sensory organs were documented by drawing with the help of a drawing attachment (Leitz) to a Leitz Dialux microscope (Leitz, Wetzlar, Germany) and later redrawn in ink. Drawings of central projection patterns were made from photomicrographs using a graphic tablet (Wacom, Kazo, Japan) and Adobe Photoshop (Adobe Systems, San Jose, CA, USA). Photomicrographs were adjusted for brightness and contrast using Corel PhotoPaint (Corel, Ottawa, Canada) or Photoshop. Figures were assembled and labelled using CorelDraw 11 (Corel) and Adobe Illustrator (Adobe Systems).

### 3.4 Nomenclature of leg nerves and nerve branches

The two main leg nerves are referred to as N5B1 (main sensory nerve) and N5B2 (main motor nerve) following Campbell [75]. The nerve branches entering these major nerves in the tibia that ultimately innervate target organs are numbered consecutively (-T1, -T2, etc.) [1].

### 3.5 Statistical analysis

The statistical analysis was carried out with Prism 4 (GraphPad, San Diego, CA, USA).

## 4. Results

### 4.1 The subgenual organ complex in *Troglophilus neglectus*

**Figure 1.**
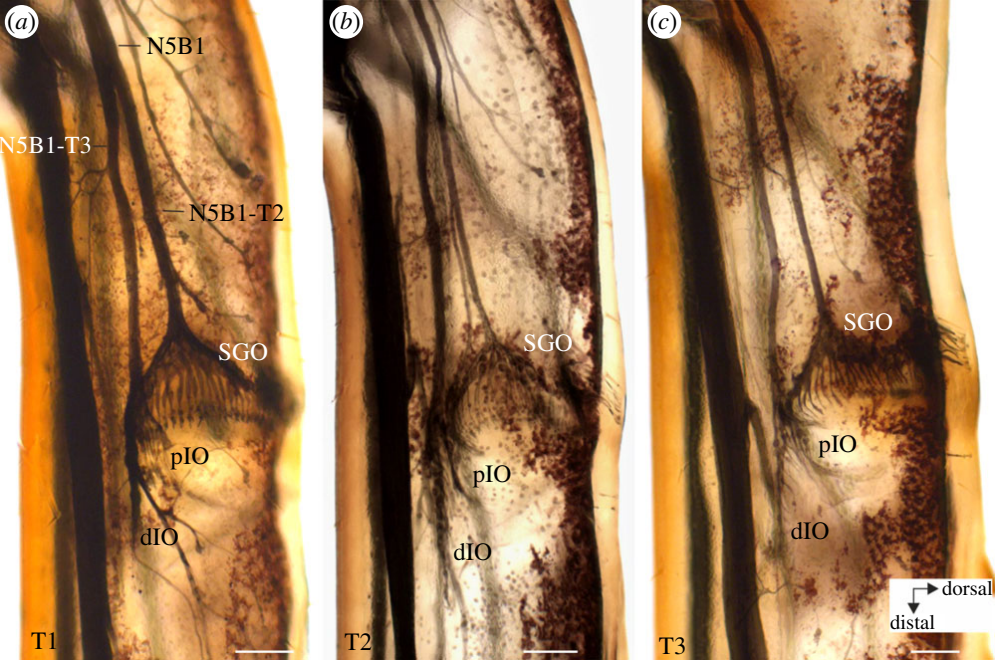
Neuroanatomical organization of the SGO complex in *T. neglectus* in wholemount preparations of the three leg pairs. Sensory elements innervated by nerve 5B1 are in focus. The SGO, the pIO and the dIO can be distinguished. Two nerve branches of N5B1 innervate the distinct organs (SGO: N5B1-T2 and IO: N5B1-T3). (*a*) T1, foreleg; (*b*) T2, midleg; and (*c*) T3, hindleg. View is from anterior. Scale bars, 100 μm.

**Figure 2.**
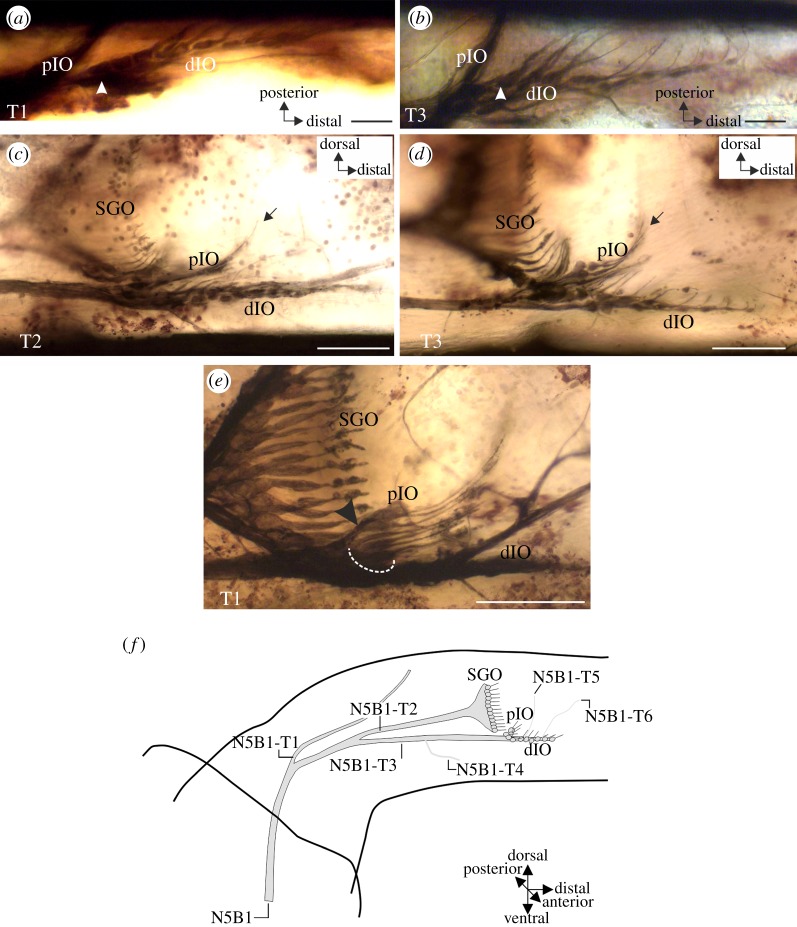
Neuroanatomy of the IO in the legs of *T. neglectus*. (*a*,*b*) Neuron somata of the sensilla in the dIO are arranged in series in a foreleg. The most proximal soma in the dIO is indicated by arrowhead. (*c*,*d*) Neurons of the pIO are positioned proximally and dorsally to the dIO. Dendrites of the pIO point dorsally with their distal segments (arrow). Neurons of the dIO extend distally into the tibia. (*e*) A section of the pIO may be supplied by a small, common nerve branch (arrowhead), as shown here from a foreleg preparation. Note that only the dorsal pIO neurons are supplied by this branch, the other pIO neurons (dotted semicircle) form no distinct nerve branch. (*f*) Drawing reconstruction of the innervation of sensory organs by nerve 5B1, showing the consensus branching pattern of the sensory nerve N5B1 in the legs of *T. neglectus*. Axes are given for the tibia. All preparations viewed from anterior. Scale bars: (*a*) 200 μm, (*b*,*c*) 100 μm, (*e*,*f*) 50 μm.

The SGO complex and its innervation in *T. neglectus* were revealed by cobalt tracing of the leg nerves (figure 1). Two nerves enter the femur: N5B1 (main sensory nerve) and N5B2 (main motor nerve). The nerve branches entering both nerves have been numbered in order of appearance from proximal to distal (figures 1*a* and 2*f*).

From N5B1, the first dorsal branch (N5B1-T1) innervates tibial sensory hairs (figure 2*f*). The next branch, N5B1-T2, supplies the SGO, and the nerve branch extending further distally is termed N5B1-T3. This nerve branch, N5B1-T3, innervates the IO. Commonly, three further nerve branches enter N5B1-T3, which all innervate hair sensilla in the tibia (N5B1-T4, -T5, -T6; figure 2*f*).

### 4.2 Neuroanatomy and innervation of the intermediate organ

The IO is exclusively innervated by N5B1 (figures 1 and 2). Sensilla in the IO can be divided into the pIO and the dIO (figures 1*a*–*c* and 2*a*–*d*). The scolopidial sensilla of these two groups lie in close proximity (figures 1 and 2). The dIO contains sensilla with the cell bodies arranged in a line (figure 2*a*,*b*). Sensilla of the dIO are located more distally of the pIO (figure 2*a*–*d*), and the dendrites of the pIO sensilla point more dorsally than those of the dIO (figure 2*c*,*d*). Dendrites of the pIO are attached to the tectorial membrane (figure 2*c*,*d*), which also covers the dIO. Somata of the pIO sensilla locate more dorsally than those in the dIO.

There are usually no distinct nerve branches separately innervating the pIO and the dIO (figure 2*c*,*d*). In a few cases, some of the pIO axons may form a minor branch before joining the nerve 5B1 (figure 2*e*). Yet, this innervation does not apply for all pIO sensilla but only the dorsal-most ones, as the ventral pIO sensilla lie at the N5B1-T3 also innervating the dIO (figure 2*e*). The minor branch was not considered a distinct nerve branch from N5B1 supplying the sensory organ because (i) it was rather short (not longer than the group of sensilla group it supplies) and (ii) it did not innervate the complete set of pIO sensilla. Similar smaller nerve branches are also present within different neuron groups of the SGO in *T. neglectus* (not shown) and may be commonly formed in scolopidial sensilla arranged along the dorsoventral axis of the leg. No distinct nerves or nerve branches usually exist for the complete set of pIO sensilla, though some variability in the innervation pattern occurs (see below).

### 4.3 Variability in the sensory innervation branching pattern

We have focused the analysis on N5B1, as this nerve innervates the anterior SGO and the entire IO. The IO consists of two anatomically recognizable sets of sensilla, while the neuronal innervation by N5B1 usually supports it as one unit of sensilla (figures 1 and 2). The most common innervation pattern by N5B1 is here referred to as a ‘consensus innervation’, which was found in 50% of preparations (figure 2*f*). Most of the variation in the N5B1 branching pattern occurred in the number of nerve branches entering N5B1 on the ventral side, which innervated hair sensilla, but not the scolopidial sense organs (for scolopidial organs, see below). Most commonly, three nerve branches occur to innervate hair sensilla (termed N5B1-T4, -T5, -T6), but a higher number occurred in a few cases (not shown).

**Figure 3.**
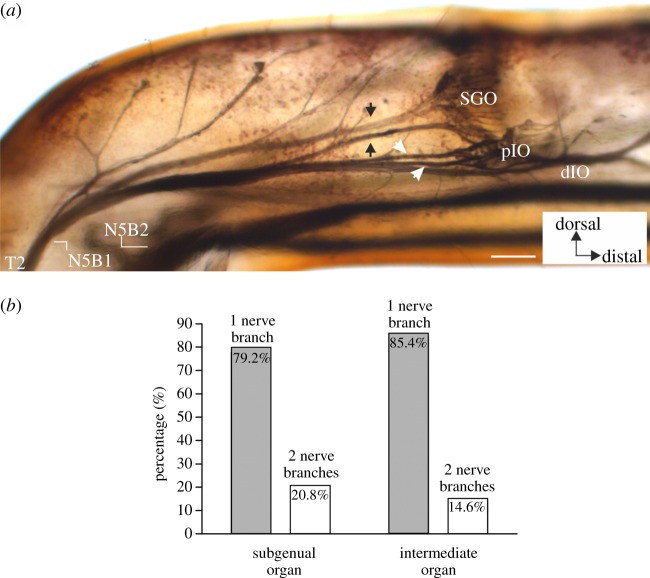
Innervation variability of the SGO and the IO from N5B1. (*a*) Midleg preparation with two nerve branches supplying each of the SGO and the IO. The two nerve branches to the SGO are indicated by arrows, the two nerve branches to the IO are indicated by white arrowheads. (*b*) Quantitative analysis of 48 leg backfill preparations. For both the SGO and the IO, innervation by a single nerve branch is most common. Innervation by a single nerve branch (grey bars) and innervation by two nerve branches (white bars). Scale bar, 100 μm.

In addition, the variability in the innervation pattern was documented for the SGO and the IO. The SGO is usually supplied via N5B1 by N5B1-T2 (figure 1). In some preparations (*n*=10 from 48), two nerve branches of N5B1 innervate the subgenual neurons (figure 3*a*). In this situation, the two SGO nerve branches are not shared with the IO. Variability in the innervation pattern is also notable for the IO: while the majority of leg preparations (*n*=41 from 48) showed innervation by a single nerve branch, N5B1-T3, in some cases (*n*=7 from 48), two nerve branches joining N5B1-T3 were innervating different parts of the IO. The branch supplying the pIO was in a few preparations longer, running very close to N5B1 (figure 3*a*). In such cases, a distinct innervation for a subset of the pIO sensilla is clearly present. In an extreme case, two long nerve branches separate from N5B1 and supply sensilla in the pIO as well as the distal pIO and the dIO (figure 3*a*).

These different innervation patterns from N5B1 were compared and quantified for both the SGO and the IO (figure 3*b*). Both organs are most commonly innervated by one distinct nerve branch each (figure 3*b*) (SGO: 79.2% and IO: 85.4%). The number of preparations with two innervating nerve branches was much lower for both organs, and the difference in proportions of innervating nerve branches between the organs is not statistically significant (*χ*^2^-test: d.f.=1, *χ*^2^=0.6433, *p*=0.4225). In the majority of preparations, both the SGO and the IO are thus innervated each by a single N5B1 nerve branch.

### 4.4 Central projections of sensory afferents

**Figure 4.**
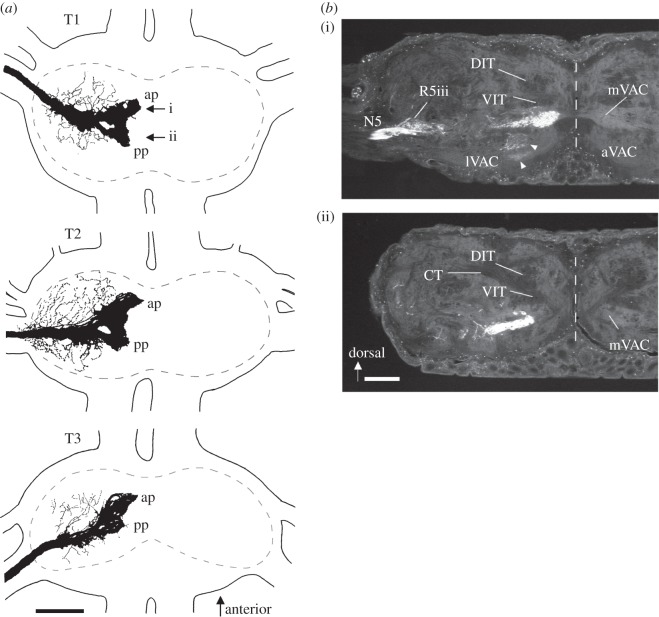
Central projection of the nerve 5B1. (*a*) Wholemount drawings of preparations from the three thoracic ganglia. The sensory afferents terminate in a dense projection close to the midline, which has an anterior projection (ap) and a posterior projection (pp). The neuropile outline is hatched. Scale bar, 200 μm. (*b*) Histological sections of a prothoracic ganglion (with the nerve 5B1 filled with Lucifer Yellow), at the level of the anterior (i) and posterior (ii) projection indicated by arrows in (*a*). Arrowheads indicate terminations in the aVAC, which are typical for sensory hairs; these projections are entirely separated from that of the SGO complex and are not included in (*a*). Scale bar, 100 μm. Midline is indicated by hatched line. T1–T3, prothoracic, mesothoracic and metathoracic ganglion, respectively; ap, anterior projections; pp, posterior projections; DIT, dorsal intermediate tract; VIT, ventral intermediate tract; CT, C-tract; N5, nerve 5; R5iii, third root of the N5; aVAC/lVAC/mVAC, anterior/lateral/median ventral association centre.

Sensory projections of the nerve 5B1 show a similar pattern in all three thoracic segments (figure 4*a*). The axon bundle enters the segmental ganglion through the slightly anterior part of the leg nerve. After giving off short processes laterally, it bifurcates medially in the neuropile into two largely separate projections. The projections are strictly ipsilateral in all thoracic segments, with the larger anterior projection terminating about 50 μm laterally, and the posterior projection about 100 μm laterally from the midline. Histological sections demonstrate axonal arborizations in the leg nerve root R5iii and the medial ventral association centre (mVAC) neuropile (figure 4*b*). Processes of the anterior projection are present in the ventral and intermediate parts of the mVAC (figure 4*b*(i)), while processes of the posterior projection are present in the ventro-lateral part of the mVAC (figure 4*b*(ii)).

## 5. Discussion

### 5.1 Sensory innervation pattern of the subgenual organ complex in *Troglophilus neglectus*

Patterns of sensory innervation have been documented in detail for legs of orthopteran insects [1,3,4]. Here, we document the innervation of the SGO complex in the cave cricket *T. neglectus*, and compare it to related insects. We paid special attention to the innervation of the IO, as previously two separate sets of sensilla were identified: the pIO and the dIO. Across Ensifera, the distinct organs in the SGO complex are typically supplied by distinct nerve branches. The aim was to study whether the pIO and dIO in *T. neglectus* have a common or distinct innervation, supporting them as one or two distinct organs, respectively. These two parts of the IO were previously distinguished based on the position of neurons' somata [41]. We found that the pIO somata are commonly advanced further dorsally but are innervated by the same nerve branch as the dIO (N5B1-T3). Such innervation was found in the majority of preparations and supports that pIO and dIO represent one single organ.

### 5.2 Interindividual variation in innervation patterns

Both the SGO and the IO may be innervated by either one or two sensory nerves (figure 3). This variability can be interpreted as structural plasticity, common in the nervous system of insects [76,77]. It may be due to stochastic developmental processes in the axonal pathfinding guided by pioneer neurons and molecular gradients during insect embryogenesis when the peripheral nervous system is organized [78–81]. In general, such structural plasticity in peripheral neural structures including the legs is greater than within the central nervous system [69,82]. Hence, interindividual differences in the innervation patterns are to be expected. In *T. neglectus*, variability in the number of innervating nerve branches was similar between the SGO and IO. Also, in both organs a situation with a single innervating branch was most commonly found. We did not find a case where both organs were innervated by a single joint nerve. A study on sensory innervation in the legs of the tettigoniid *Ephipigger ephipigger* also indicated variability in nerve branches [1]. In this case, the addition of nerve branches was prominent, while fusion of nerve branches from separate organs did not occur. This leads to the assumption that if the minimum number of nerve branches in the sensory organ complex is also most common across individuals, it may be representative for the specific species under study. Variability has previously been noted in tympanate and atympanate ensiferan species [1,39] but not quantified for comparisons.

### 5.3 Comparative innervation and central projections of the subgenual organ complex in Ensifera

The SGO complex in *T. neglectus* has been considered as ‘primitive’ compared to other, tympanate Ensifera due to its composition of the SGO and IO [41]. Among most atympanate Ensifera, the *crista acustica* homologue is an organ homologous to the tettigoniid auditory sensilla, which is the third sensory organ in the complex (table 1). We have tested for the possible presence of a *crista acustica* homologue in *T. neglectus* as a representative of the cave crickets, based on sensory innervation. While the sensilla in the dIO of *T. neglectus* are reminiscent of *crista acustica* by their linear arrangement, the neuroanatomical results on innervation of this sensory structure in *T. neglectus* do not identify it as homologous to the *crista acustica* in tettigoniids.

The innervation pattern described in *T. neglectus* is consistent with the presence of two scolopidial sensory organs, the SGO and the IO. The IO is differentiated into the proximal and distal parts based on the spatial arrangement of sensilla. Such a division is also described for tettigoniids [62,63,83] and a schizodactylid species [64]. In contrast to the situation in *T. neglectus*, the IO in the other atympanate Ensifera is innervated by a prominent IO nerve, which enters N5B1 at a more proximal position than the nerve for the *crista acustica* homologue [39,64].

Less clear are comparisons to the cricket ear where the IO was not clearly identified, but subgroups of auditory sensilla have been described in the tympanal organ [28,29,84]. These subgroups were not identified by a separate innervation [84] and cannot be recognized as distinct organs, making a direct comparison to other taxa difficult [23]. The comparison of the situation in *T. neglectus* to outgroup taxa is easier, as outlined below.

In *T. neglectus*, central projections of the axons within the nerve 5B1 form a pattern closely reflecting that of other atympanate Ensifera, especially Gryllacrididae and Stenopelmatidae [39,40]. These afferents have been described for the prothoracic segment [85], while their serial organization is documented here. In all thoracic segments, the projections lie in the medio-ventral association centre, forming a typical anterio-posterior bifurcation that terminates somewhat laterally from the ganglion midline. In the tympanate Ensifera, on the other hand, the extensive anterior projection of auditory afferents reaches the midline, so that the neuropile is bilaterally fused [4,31]. Remarkably, the general extent of the sensory projections area seen in dorsoventral view does not appear smaller in *T. neglectus* compared with other atympanate Ensifera, despite a lower number of scolopidial sensilla in the SGO complex of the cave cricket (lacking a *crista acustica* homologue). In addition, the anatomical position of the N5B1 projection in the intermediate and ventral part of the mVAC is similar between the species. This central projection pattern confirms the mVAC as a primary mechanosensory neuropile conserved in tympanate and atympanate Ensifera. The prominent projection of the SGO complex in *T. neglectus* may reflect the behavioural relevance of mechanosensory information in this species.

### 5.4 Evolution of the subgenual organ complex in Ensifera

**Figure 5.**
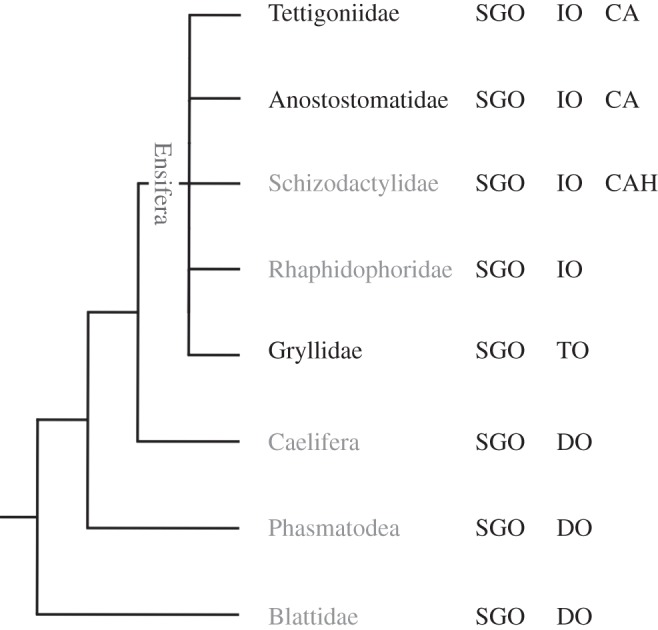
Phylogenetic relationships of orthopteroid insects. Within Ensifera, the relationships are currently not resolved. In the phylogeny, sensory organs are summarized for the respective groups. Groups that lack tympanal organs in the tibia are set in grey, mainly tympanate groups are set in black (though cases of secondary reduction occur, the majority of species has tympanal organs). CA, *crista acustica*; CAH, *crista acustica* homologue; DO, distal organ; IO, intermediate organ; TO, tympanal organ. Phylogeny adapted from Grimaldi & Engel [90].

**Figure 6.**
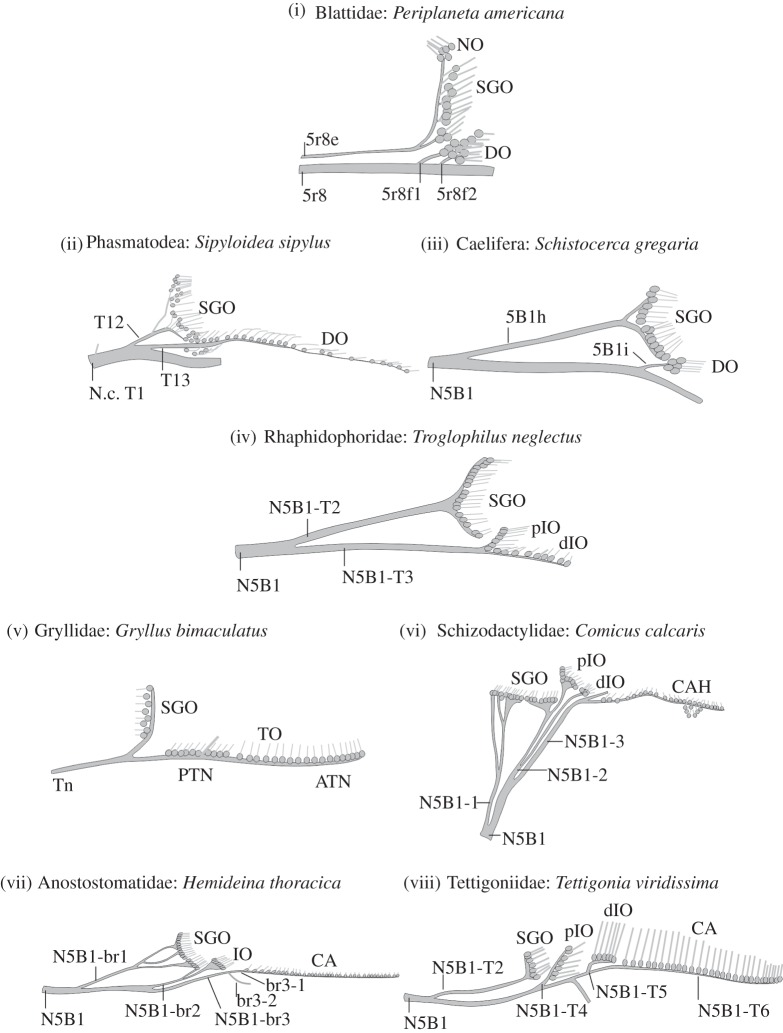
Comparison of innervation pattern in the anterior SGO complex in Ensifera and sister and outgroup taxa. Different sensory organs innervated by the anterior nerve (mainly N5B1) are recognized in the respective taxa, and terminology of the nerves and nerve branches is given as used for the respective species and is not congruent across taxa. Importantly, naming of nerves or organs does not generally address potential homologies. The nerves included innervate the SGO as well as hearing organs or their homologous structures. Based upon data from *Periplaneta americana* [22], *Sipyloidea sipylus* [21], *Schistocerca gregaria* [3], *Troglophilus neglectus* (present study), *Gryllus bimaculatus* [28,84], *Comicus calcaris* [64], *Hemideina femorata* [4], Tettigoniidae [1,23,31] and own unpublished observations (2012–2013).In Tettigoniidae, the IO may be innervated by a single nerve branch (own observations) or two nerve branches [23]. ATN, anterior tympanal nerve; CA, *crista acustica*; CAH, *crista acustica* homologue; DO, distal organ; IO, intermediate organ; N.c., *nervus cruris*; NO, Nebenorgan; PTN, posterior tympanal nerve; SGO, subgenual organ; Tn, tympanal nerve; TO, tympanal organ (Gryllidae).

For Ensifera, currently two major scenarios for the evolution of the SGO complex and of the tibial hearing organs are discussed, based on the different phylogenies suggested for this group [25,26,40,50,86]. The phylogeny of Ensifera is not resolved with conflicting relationships proposed [87,88,89] (figure 5), which hampers the reconstruction of auditory organ origins. Morphology-based phylogenies suggested that the lineage of Ensifera was ancestrally atympanate and placed Rhaphidophoridae at the basis of Tettiogonioidea [25,50,51]. A molecular phylogeny based on extensive taxon sampling derived the ancestral presence of tibial tympanal ears in Ensifera, suggesting that atympanate Ensifera underwent repeated reduction of tympanal hearing organs in several lineages [86]. The ancestral group in this molecular phylogeny are tympanate Haglidae, which possess the SGO and the IO, and the *crista acustica* [38] like tettigoniids, together with a similar innervation. If *T. neglectus* also showed remnants of the *crista acustica*, this would be consistent with the common presence of a *crista acustica* homologue in atympanate Ensifera and would also support the scenario of multiple tympanal reductions. Yet, the neuroanatomy in *T. neglectus* clearly differs from the conserved pattern of sensory organs innervated by N5B1 among other Tettigonioidea (SGO, IO, CA or CAH; figure 6 and table 1).

An ancestrally more complex sensory structure including a *crista acustica* homologue might have been hypothetically simplified in the lineage of cave crickets and *T. neglectus* in particular, resulting in a sensory complex with only the SGO and the IO clearly distinguishable. The evolutionary reduction of sensory organs is common in animals [91–95]. However, the sensory organs and the innervation pattern among Tettigonioidea seem otherwise conserved despite a variety of leg morphologies and sizes in the different groups. Furthermore, if the dIO was a remnant of the *crista acustica* that lost its distinct innervation, there is no obvious reason why any evolutionary reduction would affect sensory innervation stronger than the scolopidial sensory organ. This is also underlined by the atympanate tettigoniid *Phasmodes ranatriformis*, in which tympana are lost but the *crista acustica* and its innervation are still recognizable [96]. Furthermore, in the case of the atympanate schizodactylid *Comicus calcaris*, the fore- and midleg tibia are largely inflated and have an increased diameter, while the hindleg tibia is much thinner. Nevertheless, the only notable difference in the neuroanatomy of the complex tibial organs between leg pairs is a smaller number of sensilla in all sensory organs in the thin hindlegs, while the innervation pattern is identical in all leg pairs [64]. Still, the innervation pattern could have been secondarily simplified in *T. neglectus*, but there is no direct support for this from the sensory neuroanatomy.

The sister group to Ensifera are Caelifera, and outgroups are Phasmatodea, Blattodea and Mantophasmatodea, which have in common an SGO and a distal organ innervated usually by one nerve branch each (electronic supplementary material, table S1). Only in *Periplaneta americana* is the distal organ innervated by two nerve branches of the main nerve supplying the SGO complex [22] (figure 6). The innervation pattern present in *T. neglectus* is thus similar to the situation in the sister group of Caelifera (grasshoppers) and the outgroup of stick insects, where the SGO and the DO are supplied by one nerve branch each (figure 6). The innervation in cave crickets thus shows the pattern consistent with the plesiomorphic situation in Ensifera, despite a notable differentiation of the IO in a proximal and a distal part. These considerations imply that the DO of grasshoppers and cockroaches may likely represent the IO of Ensifera [51]. However, earlier neuroanatomical comparisons were not based on phylogenetic relationships [19] and did not correlate sensory organs by the use of a consistent terminology. Our data on the peripheral sense organs are also consistent with previous data on vibratory interneurons in the central nervous system of *T. neglectus*, which show ancestral features in morphology compared to homologous auditory neurons in crickets and tettigoniids [85].

While the neuroanatomy of sensory organs can inform discussion about their origins, the interpretation of the character state of sensory structures clearly depends on a reliable phylogeny. In future comparative studies, ideally both genetic as well as neuroanatomical information can be included to address the plesiomorphic organization and the sensory evolution of hearing organs in Ensifera.

## Supplementary Material

Supplementary Table 1: Comparative innervation of the subgenual organ complex

## Data accessibility

Quantitative data on the sensory nerves supplying the SGO complex from N5B1 can be found on figshare.com. Variability of sensory innervation in the SGO complex of *Troglophilus*: http://dx.doi.org/10.6084/m9.figshare.1050134.
