# Supplementary material for: The subgenual organ complex in the cave cricket Troglophilus neglectus (Orthoptera: Rhaphidophoridae): comparative innervation and sensory evolution
Source: R Soc Open Sci. 2014 Oct 1;1(2):140240. doi: 10.1098/rsos.140240 (PMC4448885; doi:10.1098/rsos.140240)
Supplement: Supplementary Table 1: Comparative innervation of the subgenual organ complex [file rsos140240supp1.doc]

**Supplementary Table 1**

Innervation of sensory organs by the main sensory nerve in orthopteroid insects. Since auditory organs are usually innervated by an anterior leg nerve, the nerve branches from this nerve are included. The subgenual organ is the only organ that can be innervated by a second, posterior leg nerve, resulting in a mixed innervation. Note that the nomenclature has mainly been developed for single species, rarely with clear reference to other or related species. The nomenclature for nerves is therefore not based on homology assessment between taxa, and hence homologous organs may be innervated by nerves with different numbering/ names.

Abbreviations: CA, *crista acustica*; CAH, *crista acustica* homolog; DO, distal organ; IO, intermediate organ; SGO, subgenual organ; TO, tympanal organ.

| **Species** | **Subgenual organ** | **Distal organ** | **Intermediate organ** | Crista acustica/ ***Crista acustica* homologue** | Tympanal organ | **Reference** |
| --- | --- | --- | --- | --- | --- | --- |
| Blattidae *Periplaneta americana* | 5r8e | 5r8f1, 5r8f2 |  |  |  | [1] |
| Phasmatodea *Carausius morosus*  *Sipyloidea sipylus* | N. c. T1-2  N. c. T1-2 | N. c. T1-3  N. c. T1-3 |  |  |  | [2] |
| **Caelifera**  *Schistocerca gregaria* | N5B1h | N5B1i |  |  |  | [3] |
| Ensifera Rahidophoridae  *Troglophilus neglectus* | N5B1-T2 |  | N5B1-T3 |  |  | present study |
| Schizodactylidae  *Comicus calcaris* | N5B1-1, 1-2 |  | N5B1-2 | N5B1-3 |  | [4] |
| Anostostomaridae  *Hemideina femorata* | N5B1 br1 |  | N5B1 br2-2 | N5B1 br3-1 |  | [5] |
| Tettigoniidae  *Tettigonia viridissima* | Subgenual nerve |  | 2 IO nerves | *Crista acustica* nerve |  | [6] |
| Tettigoniidae  *Ephippiger*  *ephipigger* | N5B1-T2 |  | N5B1-T4 | N5B1-T6 |  | [7] |
| Gryllidae  *Gryllus bimaculatus* | subgenual branch of tympanal nerve |  |  | posterior tympanal nerve  anterior tympanal nerve |  | [8] |

**References**:

1. Schnorbus H. 1971 Die subgenualen Sinnesorgane von *Periplaneta americana*: Histologie und Vibrationsschwellen. *Z. vergl. Physiol.* **71**, 14-48.

2. Strauß J, Lakes-Harlan R. 2013 Sensory neuroanatomy of stick insects highlights the evolutionary diversity of the orthopteroid subgenual organ complex. *J. Comp. Neurol.* **521**, 3791-3803. (doi 10.1002/cne.23378)

3. Mücke A. 1991 Innervation pattern and sensory supplyof the midleg of Schistocerca gregaria (Insecta, Orthopteroidea). *Zoomorphology* **110**, 175-187. (doi 10.1007/BF01633002)

4. Strauß J, Lakes-Harlan R. 2010 Neuroanatomy of the complex tibial organ in the splay-footed cricket *Comicus calcaris* Irish 1986 (Orthoptera: Ensifera: Schizodactylidae). *J. Comp. Neurol.* **518**, 4567-4580. (doi 10.1002/cne.22478)

5. Nishino H, Field LH. 2003 Somatotopic mapping of chordotonal organ neurons in a primitive ensiferan, the New Zealand tree weta *Hemideina femorata*: II. Complex tibial organ. *J. Comp. Neurol.* **464**, 327-342. (doi 10.1002/cne.10780)

6. Schwabe J. 1906 Beiträge zur Morphologie und Histologie der tympanalen Sinnesapparate der Orthopteren. *Zoologica* **50**, 1-154.

7. Lakes R, Mücke A. 1989 Regeneration of the foreleg tibia and tarsi of *Ephippiger ephippiger* (Orthoptera: Tettigoniidae). *J. Exp. Zool.* **250**, 176-187. (doi 10.1002/jez.1402500209)

8. Michel K. 1974 Das Tympanalorgan von *Gryllus bimaculatus* deGeer (Saltatoria, Gryllidae). *Z. Morphol. Tiere* **77**, 285-315. (doi 10.1007/BF00298805)
